# Models and Mechanisms for COVID-19 Research

**DOI:** 10.1242/dmm.049163

**Published:** 2021-06-24

**Authors:** Kirsty M. Hooper, E. Elizabeth Patton

**Affiliations:** 1 The Company of Biologists, Station Road, Histon, Cambridge CB24 9LF, UK; 2 MRC Human Genetics Unit and Cancer Research UK Edinburgh Centre, MRC Institute of Genetics and Cancer, The University of Edinburgh, Western General Hospital, Crewe Road South, Edinburgh EH4 2XU, UK

Since emerging in December 2019, the SARS-CoV-2 virus has caused over three and a half million deaths worldwide by coronavirus disease (COVID-19) at the time of writing. Despite impressive scientific progress, variants of concern continue to arise, and global distribution of vaccines and therapeutics is lacking, leading to devastating outbreaks in certain parts of the world. To combat this, the research community needs to utilise appropriate model systems to study the virus and disease. Disease Models & Mechanisms, as an open-access biomedical journal, advocates that this research, and the resulting resources that are generated, need to be accessible and shared globally.

The scientific community's response to the COVID-19 pandemic has been remarkable. Massive sequencing efforts help us track this novel virus and its variants, and a preprint has shown that rapid tests can now accurately identify up to 90% of cases within one hour (Lee et al., 2021). Treatments and practices for patients with COVID-19 have improved with clinical experience, including the use of the corticosteroid dexamethasone for patients requiring oxygen supplementation. Immense roll-outs of effective vaccines reduce COVID-19 symptoms and serious illness, with a recent preprint from Public Health England showing that the Pfizer or AstraZeneca vaccines also lower viral transmission by 40–50% (Harris et al., 2021). Owing to the timing of data collection, the majority of people included had only received one dose, and the statistics stated are for people who had received their first dose more than 21 days before. In a recent study, yet to be peer-reviewed, small overall improvements of long-COVID symptoms were also observed following vaccination (Arnold et al., 2021). Although neutralizing antibodies generated by vaccinated individuals have been less effective against emerging strains of SARS-CoV-2 – such as the Beta variant that was first identified in South Africa – most vaccines are still effective against these strains (Shinde et al., 2021). Furthermore, the US government is now supporting a waiver of patent protection for COVID-19 vaccines to enhance accessibility to vaccines worldwide.

Despite these accomplishments, several countries are still overwhelmed with the scale of the pandemic and the WHO indicates that 2021 may be a worse year for the pandemic than 2020. A devastating second wave of COVID-19 in India has peaked with at least 400,000 daily cases and 28 million total cases, as stated on the WHO COVID-19 dashboard at the time of writing. This was linked to the emergence of the Delta variant that – data from COVID-19 Genomic Surveillance tool provided by the Wellcome Sanger Institute suggests – is more transmissible than the Alpha variant, first identified in the United Kingdom. The Delta variant is spreading globally, and Nepal now has more cases than India relative to population size. With equally devastating effects, the Gamma variant has emerged in Brazil, where the WHO COVID-19 dashboard stated the death toll had passed 400,000 at the time of writing. This variant is up to twice as transmissible, and evidence indicates it may be able to evade immunity acquired from previous infections.

To fight back and prepare for future variants, the research community needs model systems to study both the SARS-CoV-2 virus and the COVID-19 disease. In this issue, Jelte van der Vaart, Mart M. Lamers, Bart L. Haagmans, and Hans Clevers discuss recent advances in lung organoids as a platform for investigating SARS-CoV-2, which can overcome the limitations of immortalized cell lines without the need for animal models (van der Vaart et al., 2021). These advances include lung adult stem cells (ASCs) or induced pluripotent stem cells (iPSCs), as well as air-liquid interface cultures. Using these complex cell models, *in vitro* studies can help delineate the mechanisms of SARS-CoV-2 infection and transmission on a cellular level, and provide a platform for screening for therapeutic inhibitors (Lamers et al., 2021). These systems open new doors to model host genetic variance in organoids by potentially using stem cells derived from individuals with relevant mutations or CRISPR-Cas9 to genetically modify organoids. Further developments also aim to model both upper and lower airways, and include them in co-cultures with immune cells.

Importantly, Clevers and colleagues emphasise that *in vitro* systems can enable rapid drug screening studies that can then be validated in the most appropriate *in vivo* model. The importance of multiple model systems is exemplified in the case of (hydroxy-)chloroquine, which was identified in cell-line screens as a potential therapeutic for COVID-19 but proved ineffective in the clinic (Boulware et al., 2020). This was due to fundamental differences of viral entry into cells within a 2D-monolayer and those within a complex multicellular system (Mykytyn et al., 2021).

Animal models are needed to study different stages of SARS-CoV-2 infection and COVID manifestation – including long COVID, as well as the demographic differences in humans, such as increased susceptibility to severe symptoms with age (Leist et al., 2020). Owing to high levels of homology with humans, non-human primates – including rhesus macaques (*Macaca mulatta*), cynomolgus macaques (*Macaca fascicularis*) and African green monkeys (*Chlorocebus aethiops*) – are considered the most-representative animal models for studying SARS-CoV-2. Despite displaying mostly mild clinical disease, a study – yet to be peer reviewed – found that high viral replication and histopathological changes can enable vaccine efficacy and safety studies in these models (Corbett et al., 2021 preprint). The Syrian hamster shows some clinical signs of infection after exposure to SARS-CoV-2, and the value of this model is exemplified in a study in which hamsters infected with the Delta variant developed more-severe disease than animals infected with other variants (Yadav et al., 2021 preprint). SARS-CoV-2 infection in ferrets induces only mild disease but, as ferrets display predominantly upper-respiratory-tract infection, they are an ideal model to study mucosal vaccines and therapeutic agents that aim to prevent upper airway infection and/or transmission (Kim et al., 2020).

**Figure:**
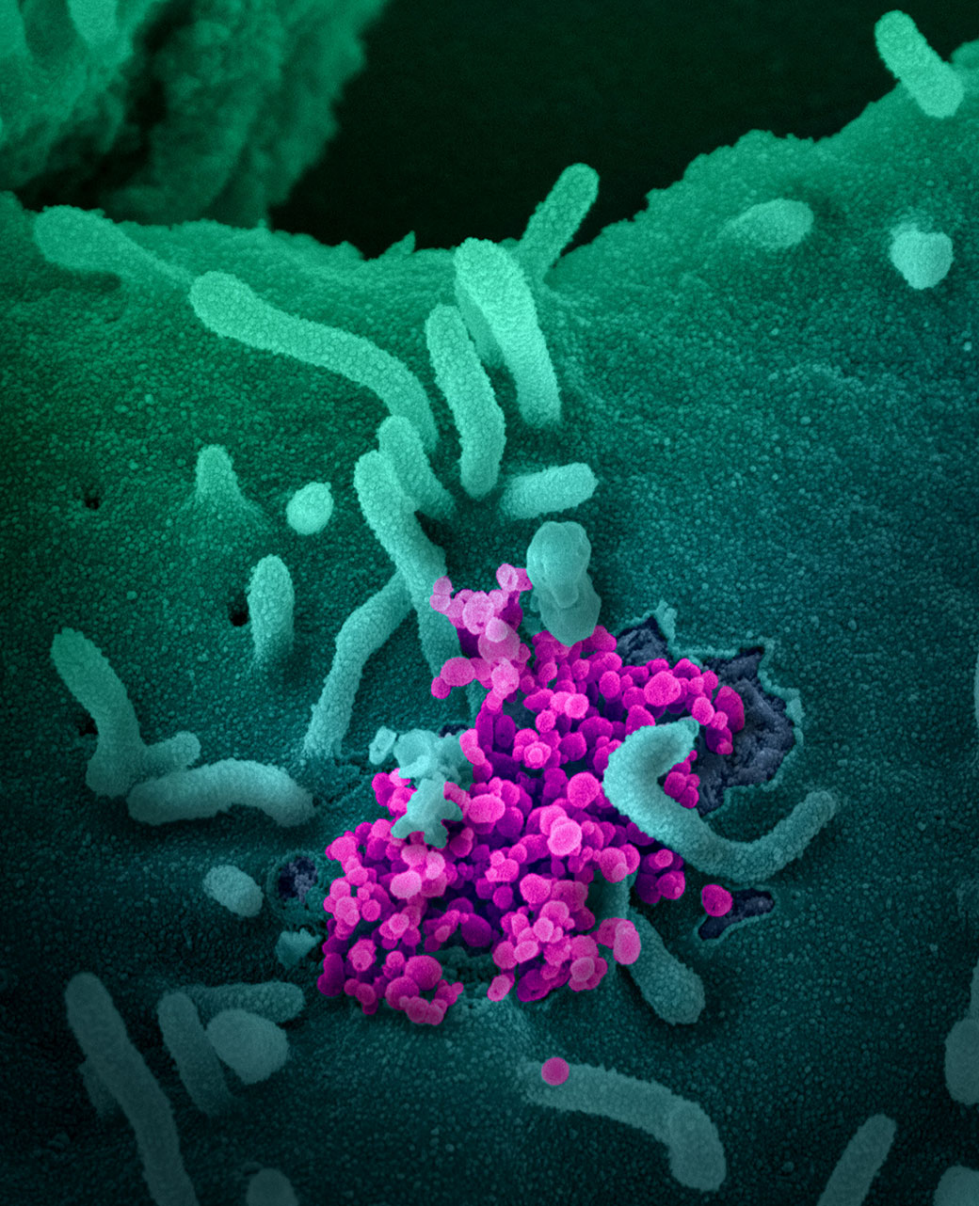
**Novel Coronavirus SARS-CoV-2.** This scanning electron microscope image shows SARS-CoV-2 (round magenta objects) emerging from the surface of cells cultured in the lab. SARS-CoV-2, also known as 2019-nCoV, is the virus that causes COVID-19. The virus shown was isolated from a patient in the U.S. This image was originally posted to Flickr by the National Institute of Allergy and Infectious Diseases (NIAID) at https://flickr.com/photos/54591706@N02/49557785757. It is reproduced without modification under the terms of the Creative Commons Attribution 2.0 Generic license.

Posing a challenge to the use of mouse models, wild-type mice are inherently resistant to SARS-CoV-2 infection because the spike protein does not effectively interact with the murine Ace2 receptor (Wan et al., 2020). Transgenic overexpression of human ACE2 can recapitulate severe COVID-19 but is also associated with lethality due to infection of the nervous system (Jiang et al., 2020). A recent technological advance has allowed for CRISPR-Cas9 generation of a human *ACE2* mouse strain in 35 days, which is a significant development as rapid model generation is pertinent in a pandemic setting (Liu et al., 2021). Mouse models can also be rapidly generated with adeno-associated virus (AAV) delivery of human ACE2; however, ectopic expression may confound the experimental system, and clinical signs of infection are mild (Hassan et al., 2020). Nonetheless, a recent study further enhanced mouse susceptibility to a mouse-adapted strain of SARS-CoV-2, by serially passaging it in mouse lungs to enable its use in mouse models where severe disease is not induced by SARS-CoV-2 infection (Muruato et al., 2021 preprint). This also enables the infection of mice that do not express human ACE2, including existing mouse models that have been genetically modified to target pathways of interest.

No single model can fully capture the manifestation of COVID-19 in humans. Indeed, some approaches are incapable of modelling specific aspects of COVID-19 but remain useful for drug screens or vaccine efficacy studies. To combat this, many studies investigating COVID-19 are now using a combination of models owing to their individual benefits. This pandemic has also encouraged investigation of viruses in non-laboratory animals to understand the transmission between species and to study zoonotic viruses before they emerge as human pathogens. A recent outbreak in a mink breeding farm, reminds us of the potential consequences of transmission between species and the relevance of studying non-laboratory animal models (Dorp et al., 2020 preprint).

With these crucial research studies emerging, the issue of accessibility of scientific discoveries and data within scientific communities and beyond has become more prominent. This has been highlighted in India, where more than 700 scientists wrote to their government requesting easier access to clinical COVID-19 data and the data from a large-scale sequencing study that is monitoring SARS-CoV-2 variants. As an Open Access journal publishing research that strives to understand and model disease, including COVID-19, DMM aims to continue supporting this global scientific effort. The power of sharing data worldwide has been proven by scientists – such as Malik Peiris and Yi Guan, whose research into Influenza A H5N1 and SARS-CoV-1 was instrumental in containing and preventing previous pandemics – and has enabled formation of world-wide pandemic strategies for SARS-CoV-2, including vaccine development. However, a critical issue remains: although we have effective vaccines, they are not sufficiently accessible worldwide and mortality among unvaccinated individuals is still remarkably high. Therefore, we need to support the development of COVID-19 disease models to continue research into therapeutics and vaccines for global distribution. Access to this progress requires us to share vaccines, resources, therapeutics and knowledge worldwide, to enable swift and well-informed responses to this rapidly changing pandemic.
